# Permanent Draft Genome Sequences for *Mesorhizobium* sp. Strains LCM 4576, LCM 4577, and ORS3428, Salt-Tolerant, Nitrogen-Fixing Bacteria Isolated from Senegalese Soils

**DOI:** 10.1128/genomeA.01154-17

**Published:** 2017-10-12

**Authors:** Nathalie Diagne, Erik Swanson, Céline Pesce, Fatoumata Fall, Fatou Diouf, Niokhor Bakhoum, Dioumacor Fall, Mathieu Ndigue Faye, Rediet Oshone, Stephen Simpson, Krystalynne Morris, W. Kelley Thomas, Lionel Moulin, Diegane Diouf, Louis S. Tisa

**Affiliations:** a Centre National de Recherches Agronomiques, Institut Sénégalais de Recherches Agricoles (CNRA/ISRA), Bambey, Sénégal; b Centre de Recherche de Bel Air, Laboratoire Mixte International Adaptation des Plantes et Microorganismes Associés aux Stress Environnementaux (LAPSE), Dakar-Bel Air, Sénégal; c University of New Hampshire, Durham, New Hampshire, USA; d Laboratoire Commun de Microbiologie IRD/ISRA/UCAD, Centre de Recherche de Bel Air, Route des Hydrocarbures, Dakar, Sénégal; e Centre National de Recherches Forestières, Institut Sénégalais de Recherches Agricoles (CNRA/ISRA), Route des Pères Maristes, Dakar, Sénégal; f IRD, Cirad, Université Montpellier, IPME, Montpellier, France; g Département de Biologie Végétale, Université Cheikh Anta Diop (UCAD), Dakar, Sénégal

## Abstract

The genus *Mesorhizobium* contains many species that are able to form nitrogen-fixing nodules on plants of the legume family. Here, we report the draft genome sequences for three *Mesorhizobium* strains. The genome sizes of strains LCM 4576, LCM 4577, and ORS3428 were 7.24, 7.02, and 6.55 Mbp, respectively.

## GENOME ANNOUNCEMENT

The genus *Mesorhizobium* was proposed in 1997 and contains almost 30 species (1, 2). These bacteria are characterized by having a growth rate that is intermediate between the fast- and slow-growing rhizobia (1). *Mesorhizobium* are phylogenetically related and distinct from the large phylogenetic group that includes *Rhizobium* and *Ensifer*. Members of the genus *Mesorhizobium* can establish a nitrogen-fixing symbiosis with legume species found in many environments, including tropical, subtropical, temperate, and arctic areas (3). This large distribution suggests their adaptation to several ecoclimatic conditions (4, 5). Many different legume species have now been studied and shown to play several ecological roles which are essential to environmental sustainability. Through biological nitrogen fixation, legumes improve soil fertility by increasing the nutrient availability, acting as pioneers, and providing protection against soil erosion (6). The ability of the legume-rhizobia symbiosis to fix nitrogen significantly reduced the use of chemical fertilizers in agriculture and limited groundwater pollution by nitrates (2).

*Mesorhizobium* strains were sampled from soils with contrasted salt concentrations in Senegal (7), illustrating a large diversity of *Mesorhizobium plurifarium* as well as new species (MSP1-3) for which several genomes have been sequenced (8). *Mesorhizobium* sp. strains LCM 4577 and LCM 4576 were isolated from rhizospheric soil surrounding a *Prosopis juliflora* plant in the Foundiougne region located in the Groundnut Basin (Senegal) in 2013 (9), while strain ORS3428 was isolated from rhizospheric soils surrounding an *Acacia senegal* tree in the Kamb region located in the sylvo-pastoral area (Senegal) in 2005 (10).

Under *in vitro* culture conditions, these strains were considered salt tolerant. Strain LCM 4577 tolerates up to 400 mM NaCl, while strains LCM 4576 and ORS3428 are limited to 200 mM. Because of these properties, these strains could potentially be used in association with leguminous plants for the reforestation of saline lands. The genomes of *Mesorhizobium* sp. strains LCM 4576, LCM 4577, and ORS3428 were sequenced to provide information on their physiology and ecology and to identify molecular markers that are involved in its tolerance to salinity. Comparative genomics of the highly salt-tolerant strain LCM 4577 with the two moderately salt-tolerant strains LCM 4576 and ORS3428 may provide insight on the molecular mechanisms involved in their tolerance to salinity.

Sequencing of the draft genomes of *Mesorhizobium* sp. strains LCM 4577, LCM 4576, and ORS3428 was performed at the Hubbard Center for Genome Studies (University of New Hampshire, Durham, NH) using Illumina technology (11). A standard Illumina shotgun library was constructed and sequenced using the Illumina HiSeq 2500 platform with paired-end reads (2 × 250 bp), which generated 8,750,732 to 19,900,494 reads (Table 1). The Illumina sequence data were trimmed by Trimmomatic version 0.32 (12) and assembled using Spades version 3.5 (13) and ALLPaths-LG version r52488 (14). Data on the final draft assemblies for *Mesorhizobium* sp. strains LCM 4576, LCM 4577, and ORS3428 are presented in Table 1. The final assembled genomes of *Mesorhizobium* sp. strains LCM 4576, LCM 4577, and ORS3428 were 7,241,525, 7,019,804, and 6,552,800 bp, respectively, with an average G+C content of 64% (Table 1). These genomes were annotated via the NCBI Prokaryotic Genome Annotation Pipeline (PGAP) and resulted in 6,665, 6,464, and 5,145 candidate protein-encoding genes, respectively.

**TABLE 1:** Genome statistics

*Mesorhizobium* strain	No. of reads	*N*_50_ contig size (kb)	Assembly size (Mb)	No. of contigs	Sequencing depth (×)	No. of CDSs^a^	G+C content (%)	Accession no.
LCM 4576	19,900,494	236	7.24	89	509.8	6,665	63.54	MDDT00000000
LCM 4577	8,750,732	305.7	7.02	56	220.3	6,464	63.74	MDDU00000000
ORS3428	16,898,886	144.6	6.55	191	477.7	5,145	63.12	MDFL00000000

a CDSs, coding sequences.

### Accession number(s).

The draft genome sequences have been deposited in GenBank under the accession numbers listed in Table 1.
